# A Narrative Inquiry Into Global Systems Change to Support Families When a Parent Has a Mental Illness

**DOI:** 10.3389/fpsyt.2019.00310

**Published:** 2019-05-08

**Authors:** Sophie Isobel, Becca Allchin, Melinda Goodyear, Brenda M. Gladstone

**Affiliations:** ^1^Research Department, Mental Health Services, Sydney Local Health District, Sydney, NSW, Australia; ^2^School of Rural Health, Faculty of Medicine, Nursing and Health Sciences, Monash University, Clayton, VIC, Australia; ^3^Eastern Health Area Mental Health Service, Melbourne, VIC, Australia; ^4^Parenting Research Centre, Melbourne, VIC, Australia; ^5^Dalla Lana School of Public Health, Centre for Critical Qualitative Health Research, University of Toronto, Toronto, ON, Canada.

**Keywords:** system change, global mental health, parental mental illness, intergenerational mental health, family mental health, children of parents with a mental illness, qualitative secondary analysis, narrative inquiry

## Abstract

The issues that confront families when a parent experiences mental illness are complex. This often means that multiple service systems must be engaged to meet families’ needs, including those related to intergenerational experiences of mental health and illness. A multisystem approach to public mental health care is widely recommended as a form of preventative intervention to address the effects of mental illness and its social, psychological, and economic impact upon parents, children, and families. Globally, a multisystemic approach to care requires a change in the way systems are currently organized to support families, as well as the way systems are interacting with families, and with each other. This qualitative secondary analysis emerged from a primary study examining global systems change efforts to support families, including components of change that were common and considered successful in different countries. A narrative inquiry method was used to re-analyze the data by compiling the stories of change described by individuals from participant countries. The data were interrogated to ask questions about story content, and to identify who was telling the story and how they described important changes across different geographical and cultural contexts. The individual stories of 89 systems change experts from 16 countries were then compiled into a shared global narrative to demonstrate international progress that has occurred over time, toward multisystemic change to support families where parents experience mental illness. While the global narrative demonstrates considerable overlap between pathways toward change, it is also important to document individual stories, as change pertains differently in different contexts. The individual stories and the global narrative illustrate how countries begin a journey toward change at different time points and may have various outcomes in mind when they commence. Study findings raise questions about the extent to which systems change can be standardized across countries that have unique social, cultural, political, and economic features. This study provides several potential points of reference for countries considering, or currently undertaking systems change to support families where a parent has a mental illness. It also provides an important story about international efforts undertaken to improve outcomes for families.

## Introduction

Mental illness represents a substantial proportion of the world’s health problems, with lifetime prevalence estimates between 18% and 36% (1), accounting for 13% of the global burden of illness (2). Globally, approximately 15–23% of children may live in families with a parent who has a mental illness (3–5). Mental illness affects families. Children who have a parent with a mental illness are at increased risk of developing their own mental health problems with approximately one third at risk of serious mental illness and another third at risk of any mental illness (6, 7). Mechanisms of risk include disrupted parent–child interactions, exposure to social disadvantage, genetic and epigenetic processes, and a lack of mediation of other risk factors (8). Risk is most likely to be transmitted through a complex interplay of neurobiological, genetic, and psychosocial factors (9). Families affected by parental mental illness can experience vulnerabilities including sustained stress and cumulative adversity (9) and are at significant social disadvantage due to factors such as poverty and social isolation (8). The effects of parental mental illness are likely comprised of bidirectional interactions between risk and protective factors at the individual, relational, community, and societal levels (10), requiring multilevel social and collective action (11). Families are affected by their environments, including a wide range of ecological factors (10), such as poverty, homelessness, interrupted education, incarceration, and political or environmental disadvantage (11). These factors can have long-standing impacts on families across generations (12).

Recovery from mental illness is also increasingly being understood to be a social and relational process occurring often within family contexts, particularly when parenting roles and children are involved (13, 14). A multisystem “whole of family” approach to mental health care has been recommended as a form of preventative intervention to address the impact of intergenerational mental illness on parents and children (15, 16). Understanding the needs, experience, and context of the whole family is of central importance to addressing parental mental illness, but is not identified to be universally well embedded in practice within any one system (4, 17). A systems approach requires partnerships beyond mental health, within education, welfare, primary health, social care, public health, and social policy development (18). Many challenges exist though at the level of the practitioner, organization, service system, and cross-service systems to provide a “whole of family” approach that meets the needs of these families. Gaps between what is known in research as best practice to promote family mental health and actual practice delivered and sustained on the ground is an issue for many countries across the world (18).

The help families do receive is most often from services that are themselves fragmented, particularly between the adult and child service systems (19). Multiple barriers prevent easy integration across service systems to meet the many needs of families. Funding for services is limited, and system barriers include a focus on the individual with the presenting issue as the point of service, rather than the family (20). A significant factor influencing systems level change is the ability for health and mental health funding arrangements to permit a broader focus on the needs of the individual with mental illness to, for example, incorporate key relationships with family members and the social context of a person’s life (e.g., parenting) (18).

Mental health promotion strategies require an intersectoral approach to implementation, as well as the use of multiple methods (21). These forms of cross sector engagement that require an integration of approaches are also known to be slow processes that unfold as a series of cumulative developments over time (21). For families where parents have a mental illness, the indication for system change comes from compounding vulnerabilities and the health, social, and economic factors that contribute to them (22). Health promotion frameworks suggest that change should be situated across all the systems that connect with families and their individual members (21).

In the context of risk to children and their families where a parent has a mental illness, Foster (23) identified that intergenerational mental illness may indeed be a “wicked problem.” This is because it is a significant social problem and complex public health issue, with multiple causal pathways and policy implications that are resistant to simple resolutions. Stories of change that have taken place or may still be unfolding in differing contexts may provide insight into global progress on the issues affecting families where a parent has a mental illness and on outcomes for these families. Such stories may also provide important guidance for service settings, including those of individual countries, who see themselves at the beginning of systems change on this topic. Rather than each country embarking on a separate or isolated journey toward change to address similar issues, a shared narrative of global change may be of benefit to those who can utilize the knowledge gained.

## Study Design

### Aim

This study aimed to construct a narrative of global systems change to support families where a parent has a mental illness through an examination of individual stories of change from countries across the world.

### Research Questions

How do individual experts in different countries describe stories of system change to support families where a parent has a mental illness?

What do individual narratives of change tell us about systems change processes globally?

### Study Data

The analysis reported in this article is a qualitative secondary analysis (QSA) of data produced in an international Delphi study examining the concept of systems change to support families where a parent has a mental illness. QSA reuses existing data, collected for prior purposes, to investigate new questions or apply a new perspective to an “old” question. An immediate conundrum in speaking and writing about QSA is the difficulty posed by language. QSA is a study in its own right; to avoid confusion, researchers generally refer to the primary study (the Delphi), as a way of distinguishing between the previous work and that to be performed in the secondary analysis (24).

### The Primary Study: An International Delphi Study Into Systems Change

The primary study was based on the experience of an international research group working in the area of families where a parent has a mental illness. For the primary study, key system change experts on the topic were identified through the networks of the research team and their professional affiliates in each of their respective countries. Individuals in each country were invited to participate in the study *via* email, followed by snowball sampling thereafter (25). Ethics approval was provided by the lead university and the affiliated recruiting service in each country where required.

Participants in the primary study included service development workers, policy makers, managers, practitioners, and health or welfare workers who had expertise at working at the system level as well as an understanding of change over time in their respective countries of work. This included psychologists, social workers, occupational therapists, mental health nurses, psychiatrists, researchers, consumer advocates, government officials, and administrators.

The first round of the Delphi study asked open-ended questions about experiences of system change within the participants’ geographical and cultural contexts. Specific systems were not specified but systems change was defined as any workforce, policy, legislation, or other mental health promotion strategy or development that aimed to identify and support parents with mental illness and their families, including their children. Participants were asked questions about: steps and approaches to systems change initiatives undertaken within their countries, locally or nationally; factors that facilitate change or remain as barriers to change; the most significant change that had occurred up to the point of the study; and considerations about future work needed to bring about systems change. The direct role of the participants in systems change was also explored as a way to understand their experience and efforts undertaken over time.

### The QSA: Developing a New Research Question About the Experience of System Change Using Narrative Inquiry

In preparation for subsequent rounds of the primary Delphi study, a thematic analysis was used to identify key categories based on data from the preliminary round of questioning addressing individual experience with systems change in each country (26). As the thematic analysis was reviewed, questions arose about how to retain the stories of change described by participants and how, or if, individual stories might fit together in order to construct a global narrative of change. A common problem identified in qualitative data analysis is that categorization can result in data fragmentation such that the “meaning of the whole” is obscured or lost altogether (27). The thematic analysis, while useful for developing further rounds of the primary Delphi study, was not designed to capture narratives of systems change because the analytic emphasis was on determining common elements related to each country’s experience as it unfolded across time and place. Consequently, narrative inquiry was chosen as a methodology for carrying out secondary data analysis, to answer a different question about “systems change,” as storied.

### Narrative Inquiry Methods

Following a modified version of Labov’s (28) narrative framework, specific questions were used to analyze and interpret the data from each participant in the primary study. Analytic questions included a focus on how the participant orients the audience to a story about systems change, asking, “What happened first?” (the orientation). This was followed by questioning “What happened next?” or “What happened once the initial change had taken place?” (the complication). “What still needs to happen?” reflected an analytic focus on understanding how stories of change might be unfolding currently, considered unfinished, and seeking resolution of some kind (the resolution). The next step in the analysis and interpretation involved compiling the individual country-by-country stories into a collective narrative (29), or broader story of global systems change.

The narratives produced in this study are based on an analysis and interpretation of data produced by participants in the primary study. Participants varied according to their country of origin, as well as the disciplinary and professional contexts (or systems) in which they worked as experts in systems change. This variation was also the case for the research team that performed the QSA. It is important to acknowledge that these contexts influence the stories that are told—those that are both proximal, local, or nearby, as well as more distant contexts, which lie outside of but influence the immediate encounter in which the data are produced, and while these stories are embedded in a wider story of change, they are not all of that story.

Reflexivity is a key analytic strategy in qualitative research because critical self-reflection (including collective team-based reflexivity) is used to question and document assumptions individuals bring to a study topic. This knowledge is accounted for and used to produce additional insight into the data during analysis and interpretation, and to acknowledge that all analysis is always partial, tentative, and provisional, and open to re-interpretation (30). In our case, understanding the significance of contexts, as part of a reflexive research strategy, provided additional insight into the complexity and subtleness of the situation under study, enriching our ability to interpret what the data might mean, and acknowledging that this is not the entire story of systems change that might be told. Reflexivity was maintained as a research practice to ensure the trustworthiness of the QSA among the research team throughout the study (31). For example, regular fortnightly data analysis meetings were used to reflexively consider and document researcher assumptions and responses to the data, consider the ongoing analysis, and interrogate and challenge emerging interpretations, including how stories were represented in writing up the analysis.

## Results

The 89 participants who responded to the Delphi study self-identified as systems change experts. They came from 16 countries, primarily from high-income countries. The majority were aged between 40 and 60 and had worked for over 10 years in their fields, suggesting a depth of experience. Most worked in public mental health services in a variety of professional roles, which is important in considering their points of reference when talking about the systems (see **Supplementary Table 1** for participant demographics). Findings are presented using the modified analytic categories of Labov’s model described previously (the orientation, the complication, and the resolution).

### What Happened First? (The Orientation)

The orientation introduces how the story of change began, according to the study participant. For example, change was described as being inspired by shifts in national policy, the introduction of an intervention model, or the creation of practice guidelines; however, it was not always apparent what motivated initial change. Sometimes change occurred, or became apparent, because it was opportunistically connected to other, related change initiatives, such as social movements associated with consumer advocacy or general mental health reform. In other instances, change could be traced back to a critical incident, such as the death of a parent or child and the system review that followed, or to the explicit efforts of a particular group of leaders who were variously described as, “pioneers,” “early adopters,” “enthusiasts,” “advocates,” or “champions” within systems change. Participants did not always situate the beginning of the story in the years that led up to change, but rather they chose a particular point in time to represent when change happened. For example, a participant described securing funding as the point of change rather than the years of lobbying that had led up to this point. Participants who had been involved in years of advocacy often went further back in time to locate the beginning of change among particular disciplinary efforts toward awareness raising, as described by a participant who stated that, “The movement began in the late 90’s where some child psychiatrists and adult psychiatrists became interested in the relationship between parental mental illnesses and children’s difficulties.”

Participants positioned stories of change within multiple types, levels, and dimensions of systems. Stories were based within local initiatives and projects, regional collaborations, or national campaigns and policies. The need for change was directed toward the individual practitioner or the wider service system and at the local, national, or international level. Participants reflected on the efforts of small motivated groups of individuals working to advocate for and drive local practice change within systems, as well as efforts to foster the government and political buy-in required to provide resources and sustain future systems change.

Although at times participants used the concept of time to orient their stories, which included notions about progress or the movement from one point of change to another, this was not consistent within or across stories. Participants varied in how they located their country’s progress toward change. For example, they described their country as being at the “beginning” of a journey, having come only recently to an awareness of what some of the issues for families in these circumstances might be. This is in contrast to others who described the journey as more of a refinement toward systems change, with already-established and multilevel buy-in from key players. How they perceived their country’s position in terms of systems change, as a temporal ordering of events, was relative and dependent on how they interpreted what progress might look like. This was the case where struggles to provide basic services within the mental health system were still considered progress. While others, who had advocated for many years in this field, and had made significant legislative changes specific to the inclusion of families in care provision, described their story as, “still having far to go.” Participants from different countries often compared themselves to other countries in taking a position and providing a rationale about their (lack of) progress on the topic, as illustrated by the following participant, who said, “We are back years with respect to other countries that have developed preventive programs. The stigma towards mental illness is still high and there is no culture of prevention.”

As individuals, participants positioned themselves in different ways within their own stories, and in the process of change that they wanted to see happen. For example, systems change was sometimes described as something external to the individual, which had to change, whether or not the participant actually worked in these systems. Participants depicted other players as more responsible for change in stories where *they* (not *I*) were the main protagonists, or the system (*itself*) was expected to change, as if it could do so devoid of human agency. This was in contrast to other stories where the pronoun “we” suggested that the participant expected to play a significant role in changing power structures for systems change to occur. The specific use of pronouns in these stories indicated how participants thought about their role and responsibility for systems change, to what extent they expected this to be an active rather than a passive role, and whether or not they saw themselves as an integral part of the change process. This is nicely illustrated in the following statement in which the participant uses the inclusive pronoun, “we,” to suggest that there is a collective responsibility to hold others to account for change that may have taken place, but has not been effectively implemented, “We need to be more hands on and make them accountable for adhering to the policies that are already in place.”

Box 1 **Orientation to the global narrative.**

Globally, systems change to improve outcomes for families where a parent has a mental illness has been initiated within differing systems and at multiple system levels.

The identified starting point of change can occur after years of action, awareness raising, and advocacy by individuals within and outside systems examining ways to improve internal and collaborative processes. Change can also begin suddenly or opportunistically in response to other pressures or actions within systems. Actions to initiate local change to practices or processes require strategic buy-in to progress toward sustainable change within or across systems.

### What Happened Next in the Stories? (The Complication)

The complication explores what happens after change is initiated in these stories. Initial change was often followed by other actions within and between organizations to drive overall systems change. For example, further change might occur through changing current organizational policies and procedures, or by establishing a network of champions to support practitioner development. Collaborative models, particularly between mental health and social care/family welfare systems were frequently sought. Some countries sought government funding to scale up the integration of evidence-based family interventions across all services working with parents. Not all countries included stories of systems collaboration as the necessary next step. Some were yet to decide on which system should take primary responsibility for supporting families, or how to collaborate with each other, as illustrated in the following quote, “There is no consensus about whether the aid [for families] should be in the health or social sector and how cooperation should take place.”

Regardless of where change first began, efforts to move in a concerted direction were questioned with respect to sustainability, with progress seemingly tenuous, comprised of fragmented examples of good work, requiring ever more resources to sustain change. “There are pockets of good practice but this is very patchy so I feel we are a long way from being able to say that there has been any significant shift in the right direction.” Initiatives were described as scattered, carried out by a small group of enthusiasts or driven by a motivated individual, and even if initially successful, were compromised by loss of funding. Aspects of change were described as not being large or cumulative enough to be sustainable.

Whether participants from different countries believed they had achieved systems change was not always clear. In some instances this lack of clarity was illustrated by the limited descriptions about what kind of “shift” was actually hoped for and how change would be recognized once it had taken place. In some cases there was a desire to move beyond simple awareness raising (about the needs of families), and the enthusiasm of advocates, toward ensuring individuals would act to achieve actual change: “It is not enough to raise awareness or gain people’s enthusiasm. They must know that it is within their grasp to make a difference.”

Even where some significant change had occurred, the need for change was characterized as unremitting because as this participant points out, once change has occurred in one area it must be followed up by a change in another area related to “the problem,” “There has been a shift from having to argue the need to work with families … to one about having to drive how to work [with families].” In another example, even in countries where systematic processes for identifying and taking responsibility for families was mandated by policy, or by law, there was difficulty bringing about the requisite change to ensure these requirements were met. Participants suggested that even when there was significant policy change in place this did not guarantee that individuals would change their practice to ensure policy was adhered to. Nearly all stories described change as a process that was ongoing, and that significant multisystemic shifts had not yet been sustained, but were desired.

Stories included specific and more general ways that change had occurred, including: seeking government funding and endorsing new legislation; implementing strategic approaches to identify families and their needs, as well as thinking about holistic approaches to care; increasing awareness of familial mental illness; and piloting new interventions aimed at family members. Desirable aspects of systems change were noted in the adjectives attributed to systems and services that were considered “trustworthy” and “honest” and had an “understanding” of families embedded within their organizational cultures. How services might work together or systems might be coordinated to address families’ needs was not detailed in the data. Stories of change over time might be best described using the metaphor of “a journey” to draw attention to change as a process, rather than suggesting it necessarily has a specific or predictable end point. This idea is illustrated in the following quote: “Change is a journey rather than a destination … the reality is that it isn’t a linear process but one that takes up and down journeys.”

Box 2 **Complicating Action in the global narrative.**

Any initial change to support families within systems is always followed by a need for more action to drive and maintain overall systems change. Difficulties identifying and responding to families continue broadly across current systems. Limited sustainable resources and a lack of cohesion about consistent approaches to support families between and within systems contribute to these difficulties. There are multiple differing pathways toward change that are effective and no identified “best way.” Fragments of change exist globally, but clarity around a shared vision to achieve sustainable systems change to support families is a desired next step.

### What Still Needs to Happen in the Stories? (The Resolution)

The resolution explores what still needs to happen to advance systems change and to sustain progress achieved to date. The resolution brings the story into the present, but also looks to the future. All stories suggested that there was much work still to be done, as summarized by the statement: “…there is still a long way to go.” This suggested an implicit idea that stories are meant to achieve resolution, but this was portrayed as far off. Often, an end point seemed to move further away the more progress the country made, as the complexities of a systems change process became more apparent. Countries with established intervention programs, models, and guidelines identified as having a long way to go, whereas those who situated themselves early in the journey described a clearer path forward.

Time frames used to describe ways forward were somewhat arbitrary and difficult to compare. Unsurprisingly, perhaps, stories were framed by the concept of progress. Progress was valued as a positive aspect of change, because it implied that journeys were moving toward some type of goal even if this was not yet defined. The end point was not necessarily explicit or shared openly. However, while stories were not always told as a linear narrative, most described “moving forward” in some way to achieve a “whole of family” or “whole of system” approach, where services worked collaboratively to provide support to all family members. Progress toward change was considered slow and ongoing but implicitly finite. “I have been working already for 25 years in this topic and it is still not finished.”

Participants were clear on what steps were required to make progress; the need for resources was frequently emphasized. A common story thread described the frustration of funding given, but later retracted. As one participant indicated, “implementing without money gets to be tiresome.” Stories proposed future steps that included; continued lobbying, awareness raising, the participation and inclusion of the voices of families, and continued funding for development of systems change initiatives. Monitoring systems were thought to also help keep the work on the agendas of local organizations and governments. University education and academic research are important for a sustained focus on the topic. Shifting toward prevention was identified as an aspiration for many, although how this is defined or might be undertaken was not described.

Legislative change was perceived to be the most ideal form of change, both by those who had experienced it, and those who wanted to achieve this, “Now we have these national recommendations but it is only recommendations … on what should or could be done and not a judicial document on what needs to be done as they have in other … countries.” Legislation was seen as necessary in order to bring other measures to enact change, such as practice guidelines and recommendations. Legislative change on its own was not enough because other changes were required to ensure the intentions of the laws were systematically implemented and the individuals responsible for carrying out these laws were suitably skilled.

The change people were working toward was broad and cross-sectorial, requiring complex solutions that go beyond changes to individual systems. Rather, change required broad cultural or paradigmatic shifts in thinking. Participants identified that cultural and social understandings, and beliefs about mental illness, influenced systems responses to families. As a result, many stories included community awareness campaigns to reduce structural barriers to support families as noted by a participant who said, “To continue to break down the stigma of mental illness so families are open to access supports.” Within and across services, participants expressed shared ideas for shifting the culture of systems to focus holistically on families, rather than individuals, and for moving the focus of funding and practice toward prevention and early intervention. As illustrated in the data, even in countries where the change process was seemingly well underway, there still remained a dominant biomedical and individual model of illness and health which determined funding and service system structures. Participant’s suggestions for creating such a shift included, piggybacking change initiatives onto other movements toward collaborative care work, trauma informed care, recovery, and social determinants of health.

Box 3 **Resolution to the global narrative.**

Current efforts toward systems change are occurring at multiple and different levels, resulting in potential reconfiguration of systems as well as change within individual systems. Sustained change will be limited without significant shifts within broader political, cultural, and economic structures toward prevention and health promotion; as well as recognizing the importance of family within models of health and illness. Globally, there is significant and broad commitment to improving outcomes for children, parents, and families and numerous strategies in place to ensure awareness and response to families within systems.

## Discussion

It is known that improving mental health outcomes cannot be achieved by changing one system alone but by engaging in collaborative practices between health, education, child protection, and social welfare systems to help shift the sociopolitical and economic determinants of mental health (2, 32). The current study illustrates the complexity of systems change to support families where a parent has a mental illness and highlights the need for coordinated action in multiple spheres for long-term sustainable change (18, 23, 33, 34). While there is a growing knowledge base of programs and strategies that can support family focused practice within systems, an approach aimed at changing the actual systems is required to ensure integrated, consistent, and intergenerational support for families (16, 18).

Underlying any systems change work are assumptions about which systems are relevant to families and that outcomes for families can indeed be improved by cumulative and progressive shifts in systems and the practices that they employ. The current study highlights diverse understandings of systems change and indicates the significance of considering what drives perceptions and recognition of the “need for change” in this field. This includes for example, a question about how “systems” are defined by different individuals (in different cultural contexts) and how or why this may be implicated in decisions about which kinds of changes are necessary to support families.

An important question raised by our narrative analysis is what is considered necessary to achieve change, and why systems change to support families is indicated. The stories in our data described legislative development as a desirable outcome and an important marker of systems change. However, despite significant and strategic but singular changes like this, a prevailing individual model of illness dominates mental health services globally and will likely continue to impede the integration of families into social and mental healthcare (2). Broad paradigmatic change in the ways we think about and practice global mental health to support families is necessary, to address the complexities of systems change described in these stories. As a result, the change process needs to be adaptable, possibly to work outside “systems,” and in ways that acknowledge that there is no one right way of doing things (35). A dynamic process to explore systems change includes challenging currently held assumptions about the topic and an iterative approach to theory and practice to systems change that occurs over time (36).

As a method of analysis narrative inquiry imposed a useful linearity on the complex storied characteristics of our study data concerning systems change. This analytical lens helped us to compile stories from individuals in different countries, and to structure these stories into a plausible narrative of global change. We do not intend to imply through this methodological choice that there is a set point of entry into stories of change, or to suggest that we know when stories about systems change are finished or complete. This caveat is reminiscent of the implementation science literature in which innovation takes place as a series of stages or phases (37). While change may be depicted as linear, in practice it often follows a variety of nonlinear, recursive, or re-iterative pathways (38), which are characterized by shocks, setbacks, and unanticipated events, similar to those described in our data (33).

Narrative analysis allowed us to see that it is difficult to determine when systems change is achieved, precisely because broader social and political contexts influence what is considered a successful outcome. These stories did suggest that simply aiming to change systems to improve outcomes for families might not be an end in itself, or enough on its own. This also has implications for thinking about how to define and measure systems change, whether or not the focus is on successful change. While systems change explored in this global narrative had no specified shared outcome related to an understanding of work to be completed or finalized in this field, it is clear from the responses of our participants that there is a global and multisystemic commitment to improving outcomes for children and families. There are large amounts of standalone and shared efforts to make progress on local, regional, or national systems change, however they may be improved upon with the resources available in different social, cultural, and geographical contexts. Further examination would be needed to track sustained benefits, to be able to understand outcomes achieved within global systems change (34) within its specific context, over time (33, 39, 40)

A recognized and well-defined “field” was necessary to identify systems change experts for the first stage of the primary (Delphi) study. However, the countries whose contributions are absent from this narrative also form an important part of the global picture of systems change. There are likely countries who have yet to begin work in this field, or who might conceptualize the problem and the solution, differently. It is hoped that providing a narrative analysis of systems change may help to guide countries who are just beginning to recognize this as a topic of concern, although their stories will likely be different. The systems change experts in this study came from a broad range of cultural and professional contexts and were shaped by the countries, cultures, and systems within which they lived and worked. They were influenced by local understandings and cultural discourses about mental health, as well as wider discourses about health and illness and what it means to be a family. These contexts form part of a global narrative of change, with each story contributing to the narrative, and many aspects not yet explored or understood. The stories responded to study questions about change over time. Consequently time was depicted as relative in these narratives, something happened and then more events occurred, but there was always something more to be accomplished. As people may experience and organize how they talk about their lives through time (29), it was not surprising that participants began their stories at point in time that made sense within their contexts. The resulting global narrative reflects the current moment in time during which this study was conducted.

To change systems there has to be a belief that systems are structural entities that do exist and are amenable to change. This belief may be a function of living in a highly resourced and privileged context that can support “systems” in a particular way. Across the globe there is scarcity of resources to meet the health and mental health needs of populations, resulting in inequitable systems to support people with mental illness and their families (2). While the capacity to include families in approaches to mental health care will certainly be impacted upon by the financial, cultural, and local processes of countries, global mental health directives maintain a strong focus on mental health promotion and prevention (41). Such a focus requires shared actions of governments, civil societies, international development agencies, and academia/researchers (41). Going forward there is a need to utilize systems change approaches that enable mutual knowledge exchange and enhance integrated understandings of global systems change. This includes overt recognition of differing notions of systems, families, and their needs across countries, cultural groups, and populations, as well as partnerships with emerging systems for mental health support in low- and middle-income countries.

While many societies may be replete with change agents, “a restless mix of individuals and organizations set on transforming the world,” their achievements have been described as “islands of success in a sea of failure” (35, p. 12). Stories in this study suggested that the longer countries undertake change, the further away success may appear. It is possible that those in the process of change for longer periods of time may identify the power dynamics embedded in policies and structures that continue to disadvantage families, and this may shed new light on further work to be done to bring about systems change. Considering why things don’t change can be an important step toward understanding global barriers to change (35). In the meantime as Green (35) argues, “We must become comfortable with ambiguity and uncertainty, while maintaining the energy and determination to succeed” (p. 28).

## Conclusion

Systems change experts around the world describe stories of change across systems that are diverse, pathways that are convoluted, and have only an incremental awareness of the need for family integration in individual service systems. The stories analyzed in this study can inform other stakeholders and countries as they embark on journeys toward sustainable change to support families where a parent has a mental illness. Systems change requires motivated and passionate individuals, opportunistic endorsement of the need for change, and sustained financial backing. Change can occur in different ways and with differing intentions about desired outcomes. The compilation of individual stories, coupled with a global narrative, allowed us to show how the complexity of this field might be addressed by focusing on family-focused practices within individual systems, as well as collaborative care practices across systems. The findings tell a story of complex change that highlights the different ways in which countries begin their journey; how their pathways overlap, and how the successes as well as the setbacks are experienced in cumulative attempts to improve health and social outcomes for families. The global narrative reminds us that there are many pathways to change and that it is important to recognize achievements along the way, including the potential to develop a shared outcome to support families. On this last point it is important to acknowledge that shared outcomes must be developed by including the voices of children, parents, and families who experience and use services within systems. They are missing from the current narrative but are integral to any evidence for developing systems change that might work better to support families.

## Limitations

The findings and the global narrative are a compilation of the perspectives of those who contributed to the study data at a particular point in time. The participating individuals from select countries were involved in the QSA because they were associated with the network of international researchers in the primary Delphi study, and because they self-identified as experts in system change in this field.

## Ethics Statement

The primary study was carried out in accordance with the recommendations of The National Statement on Ethical Conduct in Human Research, 2007, National Health and Medical Research Council Act with implied informed consent from all subjects. All subjects gave informed consent in accordance with the Declaration of Helsinki. The protocol was approved by the “Monash University” Human Research Ethics Committee.

## Author Contributions

MG leads the primary Delphi study. BG guided the QSA method. SI, BA, and MG led the data collation and early analysis. SI drafted the paper. All authors analyzed the data and contributed and edited the manuscript.

## Conflict of Interest Statement

The authors declare that the research was conducted in the absence of any commercial or financial relationships that could be construed as a potential conflict of interest.
