# Supplementary material for: A Narrative Inquiry Into Global Systems Change to Support Families When a Parent Has a Mental Illness
Source: Front Psychiatry. 2019 May 8;10:310. doi: 10.3389/fpsyt.2019.00310 (PMC6518018; doi:10.3389/fpsyt.2019.00310)
Supplement: Supplementary file 1 [file Table_1.docx]

**Table S1** *: Table of participant demographics*

| **Country** | **n (%)** |
| --- | --- |
| Sweden | 6 (7) |
| Ireland | 7 (8) |
| New Zealand | 6 (7) |
| Norway | 9 (11) |
| Australia | 7 (8) |
| Canada | 3 (4) |
| Finland | 9 (11) |
| Serbia | 6 (7) |
| Northern Ireland | 4 (5) |
| Netherlands | 3 (4) |
| England | 9 (11) |
| USA | 3 (4) |
| Italy | 1 (1) |
| Iceland | 1 (1) |
| Israel | 4 (5) |
| Denmark | 6 (7) |
| **Age** | **n (%)** |
| 20-29 | 2 (3) |
| 30-39 | 7 (9) |
| 40-49 | 25 (31) |
| 50-59 | 36 (44) |
| 60-69 | 11 (14) |
| **Gender** | **n (%)** |
| Male | 20 (24) |
| Female | 63 (76) |
| **Years of experience** | **n (%)** |
| 1-5 | 3 (4) |
| 6-10 | 9 (11) |
| >10 | 72 (86) |
| **Professional Role** | **n (%)** |
| Social Work/Psychology/ Occupational therapist | 16 (19) |
| Government advisor | 3 (4) |
| Program Manager/Service director | 18 (22) |
| COPMI/FAPMI | 6 (7) |
| Research/Academic | 10 (12) |
| Psychiatrist | 7 (9) |
| Mental Health Nurse | 7 (9) |
| Psychotherapist | 8 (10) |
| Other | 5 (6) |
| **Type of organization** | **n (%)** |
| Government agency | 4 (5) |
| Workforce or training organization | 5 (6) |
| Public Mental Health Service | 33 (41) |
| Hospital | 11(14) |
| Non-Government Organization | 8 (10) |
| Child Welfare | 1 (1) |
| University | 9 (11) |
| Private hospital/clinic | 5 (6) |
| Other | 4 (5) |
